# Supplementary material for: Understanding the Half-Life Extension of Intravitreally Administered Antibodies Binding to Ocular Albumin
Source: Pharmaceutics. 2020 Aug 26;12(9):810. doi: 10.3390/pharmaceutics12090810 (PMC7559355; doi:10.3390/pharmaceutics12090810)
Supplement: Supplementary file 1 [file pharmaceutics-12-00810-s001.pdf]

pharmaceutics

Supplementary Materials: Understanding the Half-Life Extension of Intravitreally Administered Antibodies Binding to Ocular Albumin

Simon Hauri, Paulina Jakubiak, Matthias Fueth, Stefan Dengl, Sara Belli, Rubén Alvarez-Sánchez and Antonello Caruso

Table S1. Summary of individual concentration-time data measured in rabbit plasma, vitreous (VH) and aqueous humor (AH).

Matrix	Group 1			Group 2			Group 3		
	Time (h)	Concentration (µM)	Subject ID	Time (h)	Concentration (µM)	Subject ID	Time (h)	Concentration (µM)	Subject ID
**PLASMA**	1	1.50E-05	1	1	2.69E-05	5	1	2.29E-05	9
	1	1.07E-05	2	1	1.32E-05	6	1	1.90E-05	10
	1	1.78E-05	3	1	1.34E-05	7	1	1.85E-05	11
	1	1.52E-05	4	1	8.30E-06	8	1	2.01E-05	12
	2	4.70E-05	1	2	7.59E-05	5	2	n.d.	9
	2	2.29E-05	2	2	1.96E-05	6	2	2.96E-05	10
	2	3.24E-05	3	2	3.40E-05	7	2	n.d.	11
	2	3.22E-05	4	2	4.37E-05	8	2	1.97E-05	12
	5	1.36E-04	1	5	2.09E-04	5	5	6.91E-05	9
	5	7.03E-05	2	5	1.02E-04	6	5	8.42E-05	10
	5	8.50E-05	3	5	1.41E-04	7	5	n.d.	11
	5	3.49E-04	4	5	1.12E-04	8	5	8.98E-05	12
	7	1.74E-04	1	7	3.26E-04	5	7	n.d.	9
	7	1.05E-04	2	7	1.42E-04	6	7	n.d.	10
	7	2.08E-04	3	7	1.99E-04	7	7	1.08E-04	11
	7	5.22E-04	4	7	1.30E-04	8	7	1.82E-04	12
	24	3.36E-04	1	23	2.54E-04	5	24	4.79E-04	9
	24	2.59E-04	2	23	1.94E-04	6	24	4.21E-04	10
	24	6.05E-04	3	23	1.99E-04	7	24	3.08E-04	11
	24	9.11E-04	4	23	1.81E-04	8	24	5.71E-04	12
	48	5.50E-04	1	48	2.87E-04	5	48	8.13E-04	9
	48	5.47E-04	2	48	4.27E-04	6	48	7.67E-04	10

	48	3.30E-04	3	48	2.09E-04	7	48	4.75E-04	11
	48	6.59E-04	4	48	1.85E-04	8	48	5.23E-04	12
	74	5.02E-04	1	70	4.23E-04	5	73	3.65E-04	9
	74	4.44E-04	2	70	1.09E-03	6	73	n.d.	10
	74	8.32E-04	3	70	4.04E-04	7	73	5.75E-04	11
	74	5.85E-04	4	70	2.81E-04	8	73	7.03E-04	12
	168	3.91E-04	1	168	6.06E-04	5	168	4.18E-04	9
	168	4.96E-04	2	168	3.05E-04	6	168	4.56E-04	10
	168	5.35E-04	3	168	5.23E-04	7	168	2.05E-04	11
	168	2.16E-04	4	168	4.61E-04	8	168	4.48E-04	12
	336	7.09E-05	1	336	1.35E-04	5	336	n.d.	9
	336	8.04E-05	2	336	8.69E-05	6	336	n.d.	10
	336	1.23E-04	3	336	1.04E-04	7	336	n.d.	11
	336	8.71E-05	4	336	2.45E-04	8	336	1.62E-04	12
**VH**	24	9.51E-01	1	23	8.68E-01	5	24	6.08E-01	9
	24	1.05E+00	1	23	6.43E-01	5	24	8.53E-01	9
	24	1.04E+00	2	23	1.12E+00	6	24	9.86E-01	10
	24	9.30E-01	2	23	8.38E-01	6	24	5.10E-01	10
	74	3.72E-01	3	70	6.43E-01	7	73	2.72E-01	11
	74	5.10E-02	3	70	9.01E-01	7	73	2.49E-01	11
	74	1.72E-01	4	70	6.75E-01	8	73	4.35E-01	12
	74	n.d.	4	70	9.93E-01	8	73	3.93E-01	12
	168	1.74E-01	1	168	3.70E-01	5	168	5.82E-02	9
	168	2.68E-01	1	168	4.93E-01	5	168	1.67E-01	9
	168	3.94E-01	2	168	1.12E+00	6	168	7.47E-02	10
	168	2.66E-01	2	168	1.57E-01	6	168	8.95E-02	10
	336	2.91E-04	3	336	2.06E-01	7	336	2.13E-02	11
	336	4.39E-02	3	336	2.03E-01	7	336	8.32E-03	11
	336	2.18E-02	4	336	2.77E-01	8	336	1.49E-02	12
	336	2.30E-03	4	336	1.67E-01	8	336	4.41E-04	12
**AH**	24	1.82E-02	1	23	2.41E-02	5	24	4.63E-02	9
	24	1.73E-02	1	23	1.20E-02	5	24	4.61E-02	9

24	1.49E-02	2	23	1.14E-02	6	24	3.30E-02	10
24	1.26E-02	2	23	4.11E-02	6	24	2.99E-02	10
74	1.91E-02	3	70	3.28E-02	7	73	2.95E-02	11
74	2.50E-02	3	70	3.08E-02	7	73	3.19E-02	11
74	6.60E-03	4	70	3.34E-02	8	73	2.93E-02	12
74	n.d.	4	70	3.65E-02	8	73	2.85E-02	12
168	1.52E-02	1	168	2.25E-02	5	168	5.99E-03	9
168	2.78E-02	1	168	1.91E-02	5	168	6.40E-03	9
168	3.31E-02	2	168	2.92E-02	6	168	3.89E-03	10
168	1.63E-02	2	168	7.64E-03	6	168	1.40E-02	10
336	1.57E-03	3	336	4.76E-04	7	336	1.60E-03	11
336	2.86E-04	3	336	4.03E-04	7	336	7.31E-04	11
336	1.08E-04	4	336	7.39E-03	8	336	7.02E-04	12
336	1.16E-03	4	336	8.19E-03	8	336	1.60E-04	12

n.d.: not determined.
